# Factors Influencing Cannibalistic Behavior of Tiger Puffer, *Takifugu rubripes*: Insights into Teeth and Stocking Density Aspects

**DOI:** 10.3390/biology15191766

**Published:** 2026-10-04

**Authors:** Haotian Ren, Zhijun Zhang, Yang Liu, Jiahao Liu, Chenchen Bian, Qiang Ma, Mengqing Liang, Houguo Xu, Yuliang Wei

**Affiliations:** 1 College of Fisheries and Life Science, Dalian Ocean University, Dalian 116023, China; 18622553631@163.com; 2 State Key Laboratory of Mariculture Biobreeding and Sustainable Goods, Yellow Sea Fisheries Research Institute, Chinese Academy of Fishery Sciences, Qingdao 266071, Chinabiancc@ysfri.ac.cn (C.B.); maqiang@ysfri.ac.cn (Q.M.); liangmq@ysfri.ac.cn (M.L.); xuhg@ysfri.ac.cn (H.X.); 3 Laboratory for Marine Fisheries Science and Food Production Processes, Qingdao Marine Science and Technology Center, Qingdao 266237, China

**Keywords:** tiger puffer, cannibalistic behavior, tooth clipping, stocking density, 5-HT

## Abstract

The current study explored the factors influencing the cannibalistic behavior of tiger puffer (*Takifugu rubripes*) based on different stocking densities and tooth clipping. Two separate experiments were carried out in this study. In the experiment testing different stocking densities, fish were reared in 300 L circular tanks with different initial stocking densities of 20, 40, and 80 fish per tank. In the experiment testing tooth clipping, some fish underwent tooth clipping treatment, while the other fish without tooth clipping served as the control. The growth results showed that high initial stocking density reduced growth, while tooth clipping numerically increased final body weight by 12.63% from 71.76 ± 2.54 g to 80.82 ± 3.33 g. The Kaplan–Meier survival curve showed that during the first 20 days of the feeding trial, the number of dead fish increased rapidly in both experiments. Cannibalism-related parameters (survival rate, caudal fin damage, and bite marks on the body surface) revealed that both intact teeth and high initial stocking density increased cannibalistic behavior. Fish with intact teeth or reared at high initial stocking density had increased serum glucose and/or lactate concentrations. Increased stocking density elevated the ratio of 5-HIAA/5-HT in the brain and altered the expression of *tph2* and *ddc* related to 5-HT metabolism. In addition, tooth clipping increased the level of brain dopamine. In conclusion, increasing the initial stocking density is a factor contributing to the increase in the cannibalism of tiger puffer. Tooth clipping alleviated cannibalism and tended to improve growth. Furthermore, tiger puffer may need about 20 days to re-establish their social hierarchy order when being relocated to a new rearing environment.

## Simple Summary

Tiger puffer exhibits sharp teeth and severe cannibalistic behavior during the larval and juvenile stages, which reduces yield and farm profitability. This study used two separate experiments to explore the factors contributing to intraspecific cannibalism in tiger puffer—different stocking densities and tooth clipping. We found that the basis of cannibalistic behavior in tiger puffer is the presence of sharp teeth, and tooth clipping alleviates cannibalism and tends to improve growth. Additionally, during the juvenile stage of tiger puffer, increasing the initial stocking density is another population factor contributing to the exacerbation of cannibalism. In addition to the stress response, stocking density-induced cannibalism is related to 5-HT metabolism, while clipping teeth is associated with an increased brain dopamine level. Furthermore, based on the results of the Kaplan–Meier survival curve, it appears that tiger puffer may need about 20 days to re-establish the social hierarchy order when being relocated to a new rearing environment.

## 1. Introduction

Tiger puffer, *Takifugu rubripes*, is a marine-farmed fish with a high market price that is highly favored by consumers in China, Japan, and South Korea [1,2]. However, this species has sharp teeth and exhibits severe cannibalistic behavior during the larval and juvenile stages, which significantly reduces yield and farm profitability [3,4]. Cannibalism is not unique to fish and has been documented in piglets and laying hens [5,6]. In piglets, needle teeth are often clipped by producers after birth to minimize the damage to littermates [7]. For laying hens, although beak trimming does not completely eliminate injurious pecking, this practice can reduce the rate of feather pecking, aggressive pecks to the head, and the mortality caused by cannibalism [6,8]. Similarly, clipping teeth to reduce cannibalistic behavior in tiger puffer is a common empirical method used by farmers. However, to our knowledge, few scientific experimental data confirm that tooth clipping can indeed mitigate the cannibalism in tiger puffer. Therefore, evaluating its effectiveness can provide scientific guidance for farmers engaged in tiger puffer culture.

For intensive aquaculture, stocking density is one of the key management strategies because it directly influences on fish growth [9,10]. Furthermore, high stocking density may reduce survival rates through intra-cohort cannibalism and aggressive interactions [11,12]. Previous studies in aquaculture have documented that many farmed fish species exhibit serious cannibalistic behavior, such as African catfish (*Clarias garipinus*) [13], striped murrel (*Channa striata*) [14], Asian seabass (*Lates calcarifer*) [15], pikeperch (*Sander lucioperca* L.) [16], and greater amberjack (*Seriola dumerili*) [17]. Cannibalistic behavior in these species occurs predominantly during early life stages and poses a significant challenge for their farming operations [18]. Such behavior can inflict injuries on fish, making them more susceptible to bacterial infections and potentially leading to mass mortality [17,19]. Consequently, optimizing stocking density is an essential prerequisite for enhancing the survival rates of piscivorous fish [20]. Therefore, it is imperative to study the relationship between fish cannibalism and stocking density. Additionally, even if aggression does not result in mortality, damage to the body surface, such as the caudal fin, will diminish their economic value [21]. Hence, it is warranted to use proper methods to mitigate or prevent cannibalistic behavior in cultured fish [22].

Due to the fact that tiger puffer exhibits cannibalistic behavior under rearing conditions, it can serve as a model species for understanding the phenomenon of fish cannibalism [23]. Currently, the literature on cannibalism in tiger puffer mostly focuses on growth, survival rate, bite marks on the body surface, and the degree of caudal fin loss [2,24,25]. However, it is not clear how the cannibalistic behavior of tiger puffer affects physiological stress and key neurotransmitters, including biogenic amines. Specifically, serotonin (5-hydroxytryptamine, 5-HT) and dopamine are known to play pivotal roles in behavioral regulation and physiological responses [26,27]. In the present study, in order to explore the factors that contribute to intraspecific cannibalism of tiger puffer and understand its characteristics, different stocking densities and tooth clipping were investigated in two separate experiments. Cannibalism-related parameters, stress responses, and the mRNA expression of key genes and metabolite levels involved in 5-HT and dopamine metabolism were analyzed in this study. It should be emphasized that these measurements provide correlative evidence linking neurotransmitter metabolism to cannibalistic behavior, rather than demonstrating a causal relationship, since no receptor antagonist or agonist experiments were performed.

## 2. Materials and Methods

### 2.1. Fish and Experimental Design

**Figure 1:**
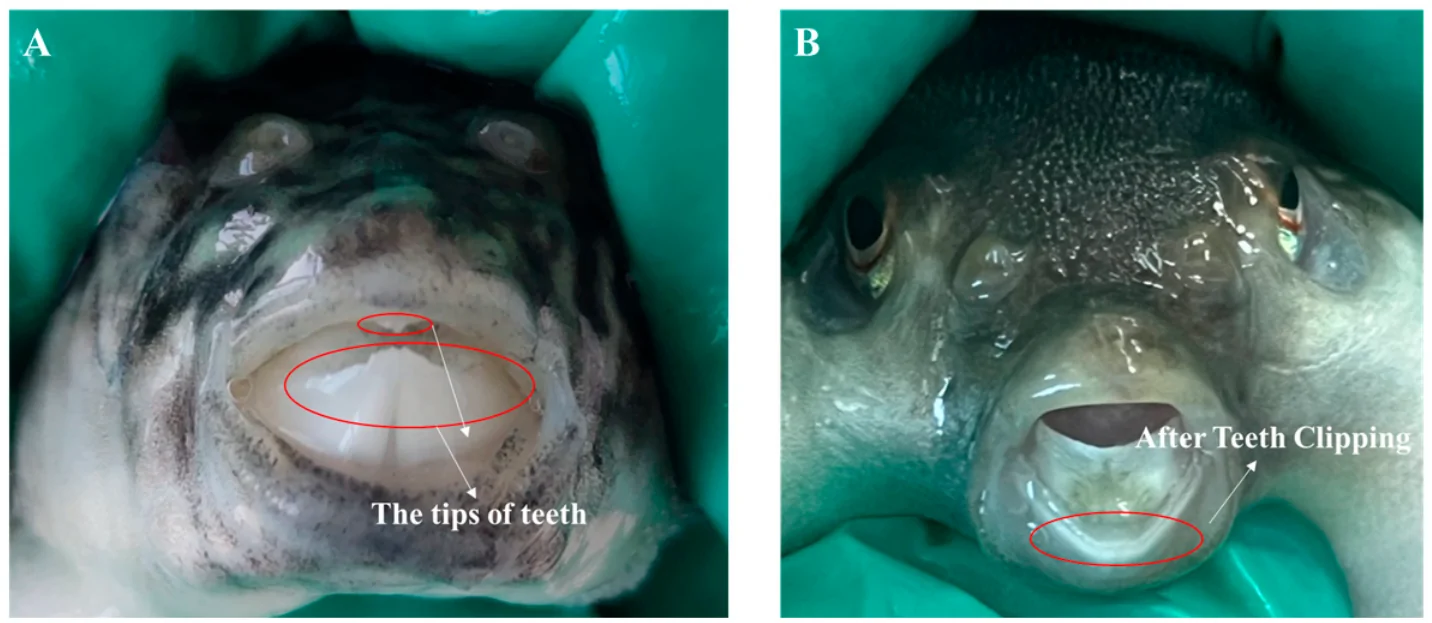
Example images of tooth clipping treatment in tiger puffer. (**A**) presents intact teeth with the tips; (**B**) presents the tips of the teeth that were partially clipped.

To explore the factors contributing to intraspecific cannibalism of tiger puffer, different stocking densities and tooth clipping were used in two separate experiments. This study was carried out at the Center for Marine Fisheries Science Research of Yellow Sea Fisheries Research Institute (Qingdao, China). Experimental fish were purchased from a fish farm (Tangshan, China). In the experiment using different stocking densities, tiger puffer (initial body weight: 7.18 ± 0.12 g) were stocked in circular tanks (water volume 300 L) with initial stocking densities of 20, 40, and 80 fish per tank (0.47, 0.93, and 1.86 g/L). Three groups were designated as stocking density—low (SDL), stocking density—medium (SDM), and stocking density—high (SDH). The stocking density trial lasted 77 days. In the experiment testing tooth clipping, all fish were anesthetized using tricaine methanesulfonate (MS-222, 30 mg/L) for the procedures (tooth clipping or air exposure). Then, some fish underwent tooth clipping treatment, in which the tips of the four beak-shaped teeth (two on the upper jaw and two on the lower jaw) (Figure 1) were partially clipped (TC). This procedure lasted approximately 30 s. The remaining fish without tooth clipping (NT) served as the control and were subjected only to air exposure for a duration close to that of the clipped tooth treatment. Afterward, all fish were acclimatized for two weeks to eliminate stress caused by tooth clipping or air exposure. The feeding trial for the tooth clipping experiment lasted 48 days. In the experiment testing tooth clipping, fish with an initial body weight of 24.28 ± 0.16 g were reared in the same 300 L water volume tanks, and the initial stocking density was 1.62 g/L (20 fish per tank). Fish in the two experiments were reared in a recirculating aquaculture system, and approximately 50% of the water in the system was replaced daily with filter-sterilized new seawater. The randomization procedure and tank allocation scheme for the two experiments were as follows: all tanks were first numbered, and each treatment was then allocated to tanks using random numbers generated in Microsoft Excel. To avoid confounding by spatial position, the allocation order was adjusted when consecutive tanks were assigned the same treatment, so that treatments were evenly distributed across the culture system. Each treatment from the two experiments had three independent replicate tanks (*n* = 3). Throughout the entire experiment, fish were kept under a natural photoperiod and fed a commercial feed with a proximate composition of 55% of crude protein, 8% of crude lipid, and 3% of crude fiber provided by the manufacturer. Fish were hand-fed four times daily (7:30, 11:30, 15:30 and 19:30) to approach apparent satiation. Measurements of dissolved oxygen, temperature, pH, and salinity of seawater were conducted weekly. The dissolved oxygen ranged from 6.5 to 8.0 mg/L, the water temperature ranged from 22.2 to 30.0 °C, the salinity ranged from 27 to 29, and the pHs ranged from 7.5 to 8.0.

### 2.2. Sample Collection

#### 2.2.1. Fish Cannibalism

**Figure 2:**
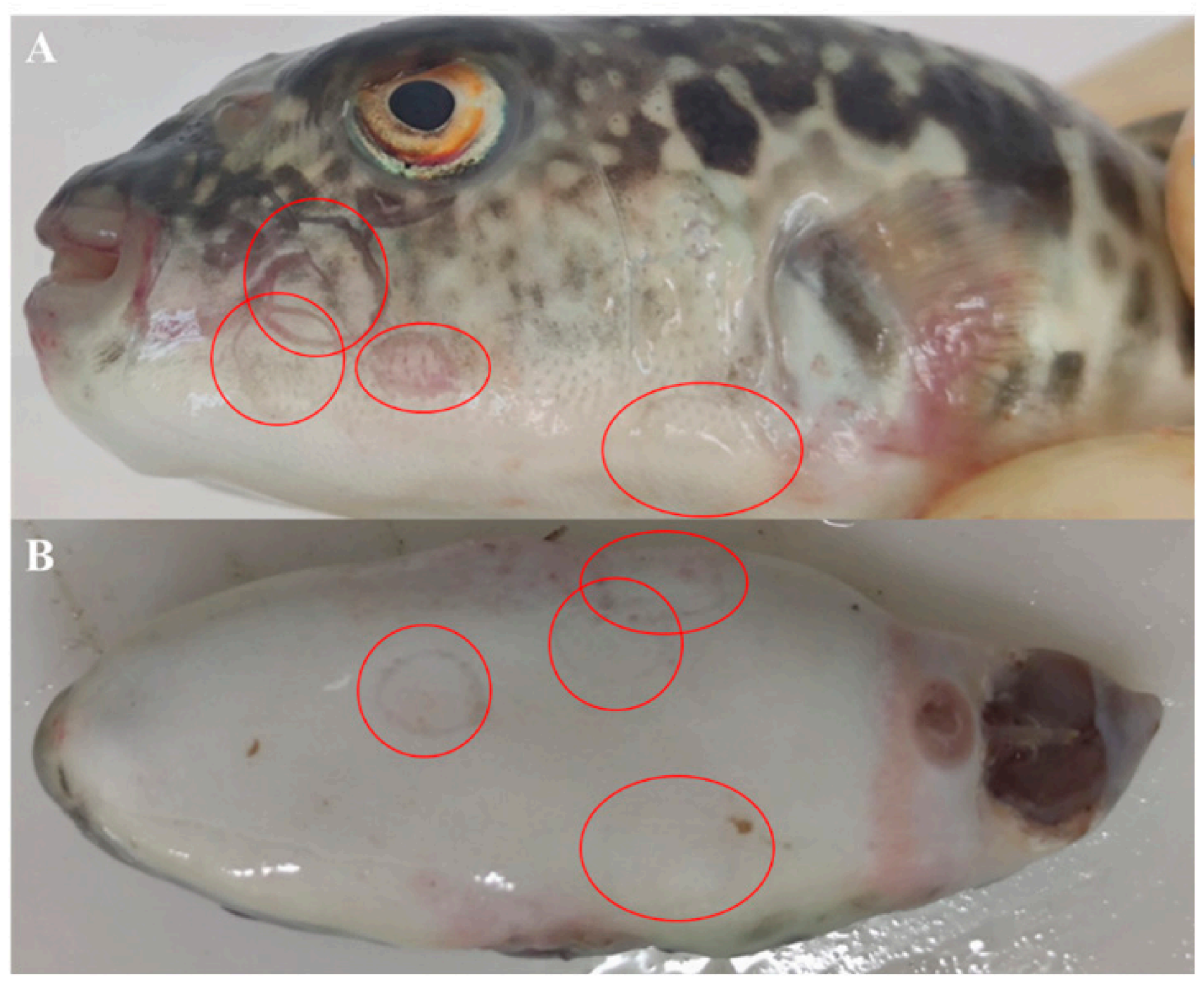
Round imprints of bite mark on the fish body surface. The red circles represent bite marks on the surface of the fish that can be counted. (**A**) indicates the lateral side of the fish; (**B**) indicates the ventral side (belly) of the fish.

**Figure 3:**
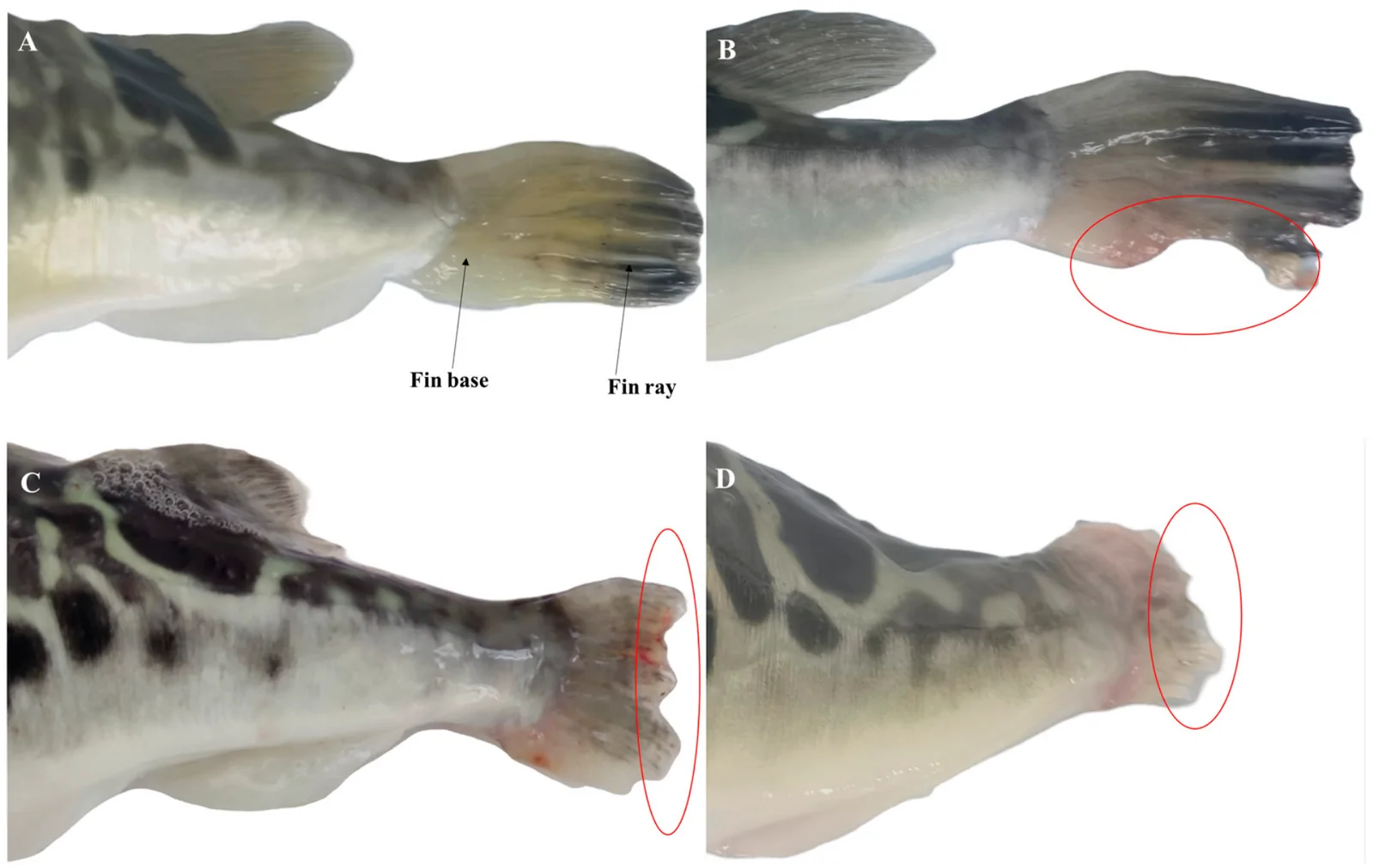
Grade 0/1/2/3 of caudal fin damage in tiger puffer. (**A**): Grade 0 represents an undamaged caudal fin with complete fin rays. (**B**): Grade 1 represents slightly damaged caudal fin with relatively intact fin rays. (**C**): Grade 2 signifies that the caudal fin is visibly damaged, with most of the fin rays becoming shorter. (**D**): Grade 3 signifies that the caudal fin is severely damaged and all of the fin rays have been bitten off.

Fish cannibalism was assessed by examining the damage to the fish body surface based on a round imprint of a bite mark (Figure 2), and to the fish caudal fin based on an ordinal scale from grade 0 to 3 (grade 0 represents that the caudal fin of fish does not have any damage, grade 3 represents the damage up to the caudal fin base) (Figure 3). Bite mark of body surface and grade 0/1/2/3 of caudal fin damage from dead fish during the feeding trial and/or surviving fish at the end of the feeding trial were individually recorded to investigate the effect of stocking density and tooth clipping on intra-cohort cannibalism of tiger puffer. Severity grades ranged from 0 to 3, which indicated that the caudal fin damage of tiger puffer increased as the number increased. In this study, all caudal fin grading scaling (Grade 0–3) was performed by the two authors, who had been familiarized with the grading criteria (Grade 0–3) using representative images (Figure 3) prior to the scoring process. To minimize intra-observer variability, the scoring was conducted in a randomized order and repeated twice for each sample, with the final score being the consensus value.

#### 2.2.2. Serum and Tissue Sample Collection and Preservation

At the end of the feeding trial, surviving fish from each tank were anesthetized by using MS-222 (30 mg/L), and then weighed and counted after completing the record of bite marks and caudal fin damage. The fish were re-fed normally for 3 days to allow physiological recovery from the possible stresses of the sampling operation described above. Then, fish were fasted for 24 h and then anesthetized again. Blood samples of ten fish from each tank were collected from the caudal vein using a 1 mL sterile disposable syringe with the needle pre-rinsed with heparin sodium to prevent clotting during blood collection. The blood samples were then transferred into a 1.5 mL plain tube (without anticoagulant) and allowed to clot at 4 °C for 4 h. The blood samples were centrifuged (836× *g* at 4 °C for 10 min) to collect the serum. After blood sampling, brain and liver tissues were collected and stored at −80 °C for subsequent analysis.

### 2.3. Measurement of Growth, Survival Rate, and Body Condition Indices

The parameters of growth, survival rate, and body condition indices were calculated as following:

Weight gain (WG, %) = 100 × (final weight of fish body − initial weight of fish body)/initial weight of fish body.

Specific growth rate (SGR, %/d) = 100 × [ln (final weight of fish body) − ln (initial weight of fish body)]/feeding days.

Survival rate (%) = 100 × (final fish number/initial fish number)

Hepatosomatic index (HSI, %) = 100 × liver wet weight/the weight of fish body

Viscerosomatic index (VSI, %) = 100 × visceral wet weight/the weight of fish body

### 2.4. Biochemical Analysis of Serum and Brain Tissue

Biochemical indexes were determined using commercial kits, purchased from Nanjing Jiancheng Bioengineering Institute (Nanjing, China), and the steps in the commercial kit protocols were followed. Liver glycogen content was measured using the Liver/Muscle glycogen assay kit (cat# A043-1-1), which was prepared as a glycogen assay solution by alkaline hydrolysis prior to measurement. Liver lactate content was measured using the Lactic acid assay kit (cat# A019-2-1); liver tissue samples were diluted and homogenized with saline solution in a 1:9 ratio prior to measurement. Serum lactate and glucose contents were determined directly using the Lactic acid assay kit (cat# A019-2-1) and Glucose assay kit (cat# F006-1-1), respectively, without additional pre-treatment of serum samples. Serum cortisol content as well as brain hydroxytryptamine (5-HT), 5-hydroxyindoleacetic acid (5-HIAA), dopamine (DA) and norepinephrine (NE) contents were measured using the method of enzyme-linked immunosorbent assay (ELISA) by their respective commercial kits (Cortisol assay kit, H094-1-2; 5-Hydroxytryptamine (serotonin) assay kit, H104-1-2; 5-hydroxyindoleacetic acid assay kit, H411-1-2; Dopamine assay kit, H170-1-2; Norepinephrine assay kit, H096-1-2). For brain samples, tissues were mixed with phosphate buffer solution (pH 7.4) at a ratio of 1:9, homogenized, centrifuged (876× *g*, 20 min), and the supernatant was collected for analysis. Optical density (OD) was measured using a microplate reader (Infinite M200 luminometer, Tecan, Männedorf, Switzerland). In this study, serum and brain samples were pooled from 10 individual fish per tank for each biochemical measurement. Samples were run with standard curves and two technical replicates to ensure quality.

### 2.5. Gene Expression Analysis

Total RNA from brain tissues was extracted using the AG RNAex Pro RNA extraction reagent (cat#AG21102, Accurate, Changsha, China). RNA quantity and purity were measured using a Colibri micro-volume spectrophotometer (Titertek-Berthold, Pforzheim, Germany). The A260/A280 ratios of all RNA samples ranged from 1.8 to 2.0, which was suitable for subsequent analysis. RNA integrity was assessed based on 28S and 18S ribosomal bands. cDNA was synthesized via a two-step reverse transcription process using an Evo M-MLV RT Mix reagent kit with gDNA Eraser (cat#AG11705, Accurate, Changsha, China). No-template controls (NTC) and no-RT controls were included in each run, and both showed no amplification and had Ct > 35, confirming the absence of contamination and genomic DNA amplification. Primers for the reference and target genes were designed on the NCBI website (Table 1) and synthesized by Qingke Biotechnology (Beijing, China). Quantitative real-time polymerase chain reaction (qRT-PCR) was prepared in a total volume of 10 μL, consisting of 5 μL of 2×SYBR mix (SYBR Green Pro Taq HS Premix II (cat#AG11702), Accurate, Changsha, China), 1 μL of cDNA (20 ng), 0.3 μL of upstream primer (10 μmol/L), 0.3 μL of downstream primer (10 μmol/L), and 3.4 μL of nuclease-free sterile water. The qRT-PCR reaction was performed on a Roche LightCycler 96 system (Roche, Basel, Switzerland) with the following program: 95 °C for 30 s, followed by 40 cycles of 94 °C for 5 s and 60 °C for 30 s. Melt curve analysis was conducted after the amplification phase (from 65 °C to 97 °C at a rate of 6.4 °C/min). Amplification efficiency was calculated using the formula E = 10^−1/slope^ − 1, ensuring that the amplification efficiency for all primers was between 90% and 110%. The relative expression level of the target gene was calculated using the 2^–ΔΔCt^ method, with ribosomal proteins L13 (*rpl13*) and L19 (*rpl19*) serving as the reference genes (the geometric mean of the Ct values of two reference genes for normalization) (Table 1) [28]. Ct values of both *rpl13* and *rpl19* across treatments are presented in Table S1. In this study, brain samples were pooled from 10 individual fish per tank for each gene expression analysis. For each treatment, three biological replicates were used for gene expression analysis. Each biological sample was run in two technical PCR replicates, and the mean Ct value was used for subsequent calculations.

### 2.6. Statistical Analysis

Statistical analyses of all data were done using SPSS 25.0 (IBM company, Chicago, IL, USA) and GraphPad Prism 9.0.0 (GraphPad; San Diego, CA, USA). Data expectations for survival curves and bite marks of body surface were expressed as mean ± standard error of three replicate tanks. Bite marks of body surface from the two experiments were presented using Tukey-style whiskers. For the experiment testing different stocking densities, one-way ANOVA test was used, followed by Tukey’s honest significant difference (HSD) post hoc test for multiple pairwise comparisons. For the experiment testing tooth clipping, independent sample *t*-test was used to compare the two groups. The *p*-value was less than 0.05, which was considered to indicate significant differences between treatments. In addition, survival curves of two experiments were analyzed by the log-rank test using the Kaplan–Meier method.

## 3. Results

### 3.1. Growth and Body Condition Indices

For the experiment testing different stocking densities, the growth and body condition indices were significantly (*p* < 0.05) affected by stocking density (Table 2). The treatments with initial 20 (SDL) and 40 (SDM) fish per tank did not differ significantly in final body weight, weight gain, specific growth rate, viscerosomatic index and hepatosomatic index, but fish in the two treatments had significantly (*p* < 0.05) higher parameters than those in the treatment with initial 80 (SDH) fish per tank. For the experiment of tooth clipping, tooth clipping resulted in a 12.63% (*p* > 0.05) increase in final body weight from 71.76 ± 2.54 g to 80.82 ± 3.33 g (*p* > 0.05) (Table 3). Similarly, compared with tiger puffer with intact teeth, weight gain and specific growth rate in the tooth clipping treatment increased from 195.57 ± 11.82% to 234.18 ± 19.26% (*p* > 0.05) and from 1.45 ± 0.05%/d to 1.56 ± 0.08%/d (*p* > 0.05), respectively.

### 3.2. Survival Analysis

**Figure 4:**
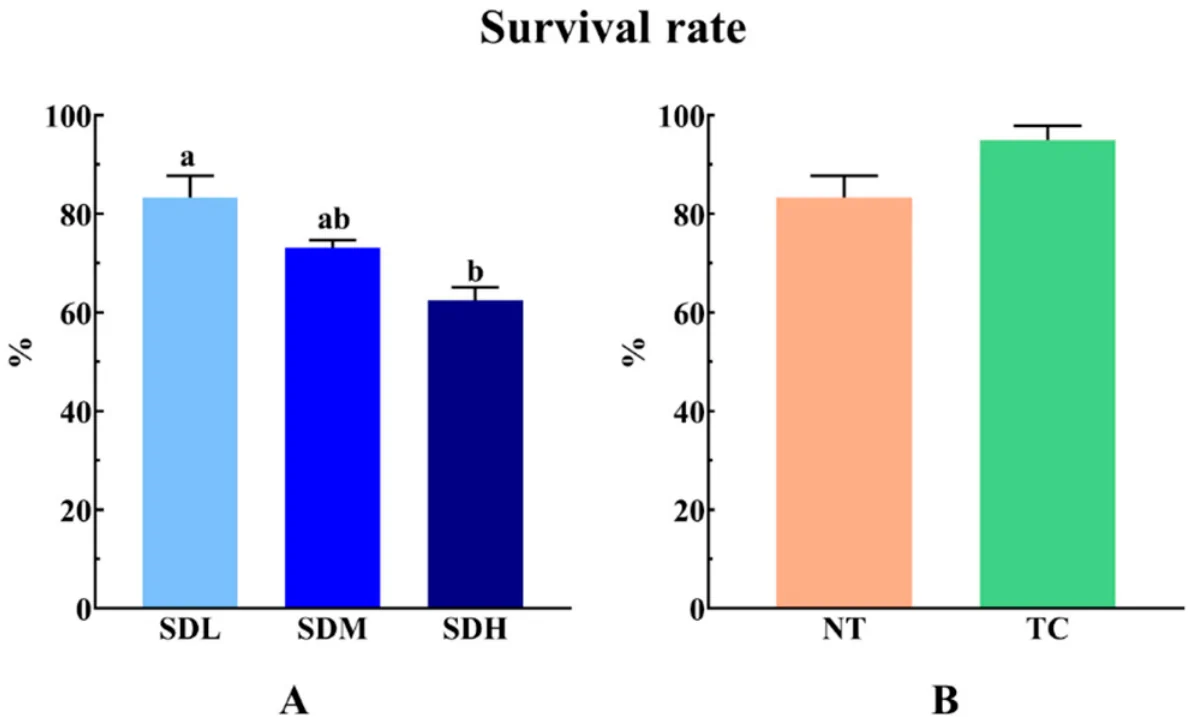
Survival rate of tiger puffer at the end of the feeding trial. (**A**) represents survival rate of the experiment of different stocking densities. (**B**) represents survival rate of the experiment of tooth clipping. Survival rate is presented as mean ± standard error of three replicate tanks. Bars with different letters are significantly different (Tukey’s test, *p* < 0.05) for the experiment of different stocking densities and *t*-test for the experiment of tooth clipping.

**Figure 5:**
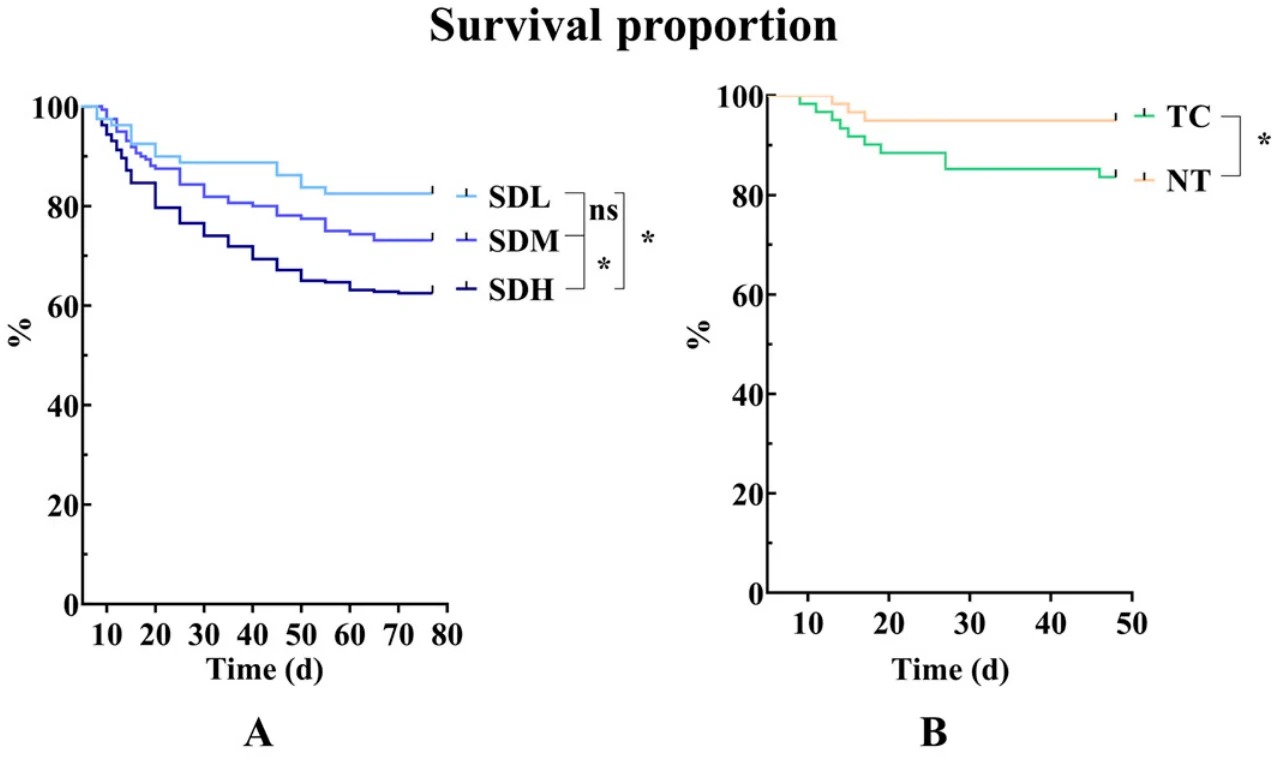
Survival curves of tiger puffer over time in the feeding trial. (**A**) represents survival proportion of the experiment of different stocking densities. (**B**) represents survival proportion of the experiment of tooth clipping. Kaplan–Meier survival curve was used to estimate survival proportion between treatments. Daily fish deaths were recorded throughout the feeding trial. “*” means significant differences (*p* < 0.05) between treatments based on Log-rank (Mantel–Cox) test. “ns” means no significant difference (*p* > 0.05).

Survival rates at the endpoint of the feeding trial are presented in Figure 4. Survival rate of tiger puffer decreased with the increase in initial stocking density, where the highest rate was found in the SDL group, which was significantly (*p* < 0.05) higher than that in the SDH group. Meanwhile, although no significant difference (*p* > 0.05) was observed, tooth clipping increased the survival rate of fish from 83.33 ± 4.41% to 95.00 ± 2.89%. For the two experiments, the results of Kaplan–Meier survival curves showed that the amount of dead fish increased rapidly in the first 20 d after the start of the feeding trial, and the survival curves overlapped partially (Figure 5). The log-rank test comparing three curves of the experiment testing different stocking densities or two curves of the experiment testing tooth clipping indicated that the survival was significantly different between the SDL and SDH groups (*p* = 0.001)/the SDM and SDH groups (*p* = 0.017) or between the TC and NT groups (*p* = 0.045).

### 3.3. Bite Marks of Body Surface and Caudal Fin Damage

**Figure 6:**
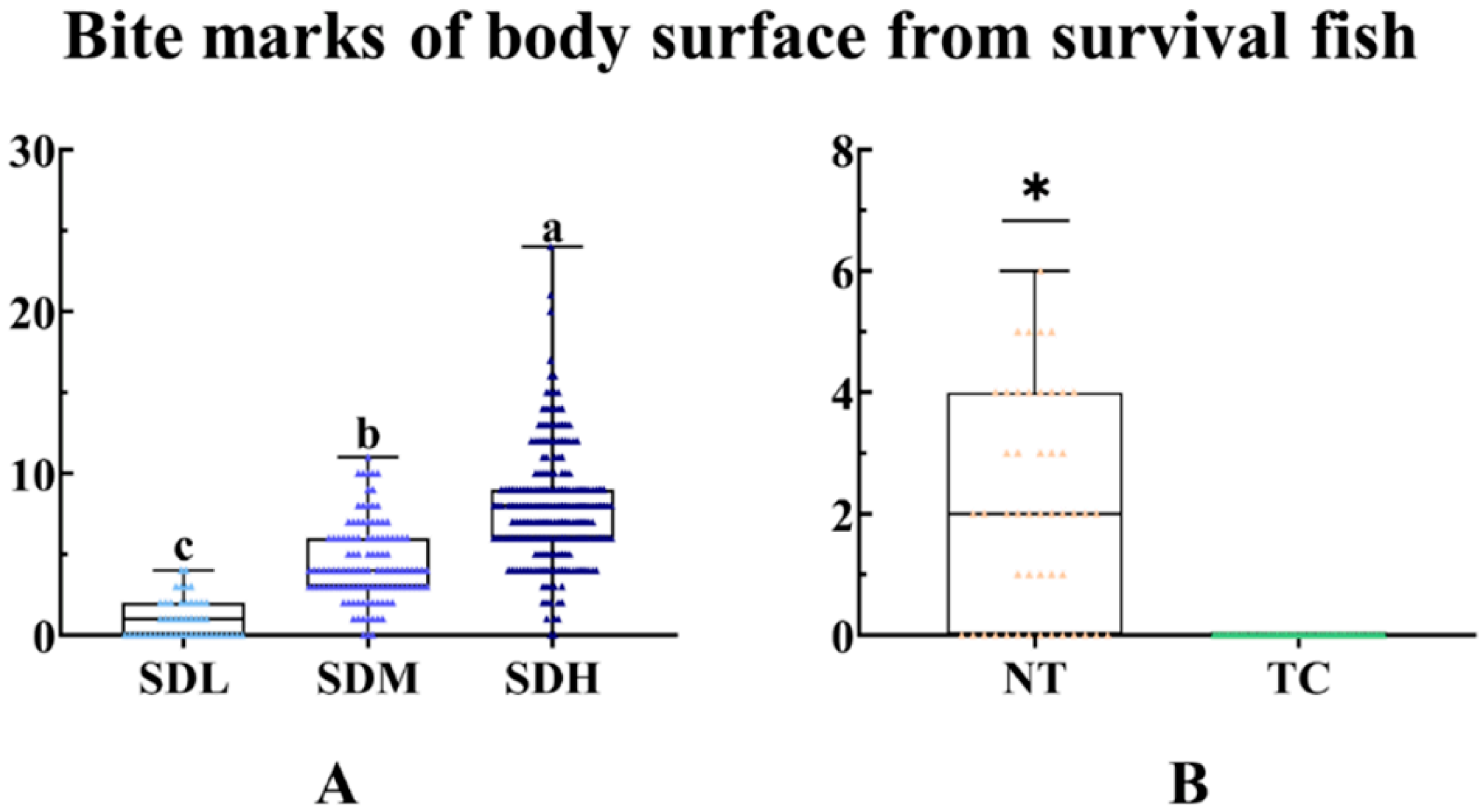
Bite marks of body surface from surviving fish at the end of the feeding trial. (**A**) is for the experiment of different stocking densities; (**B**) is for the experiment of tooth clipping. Tukey-style whiskers indicate minimum and maximum numbers of bite marks, while boxes indicate Q1, median, and Q3 values for interquartile range. Different letters are significant differences (Tukey’s test, *p* < 0.05) between treatments. “*” indicates significant differences (*t*-test, *p* < 0.05) between treatments.

**Figure 7:**
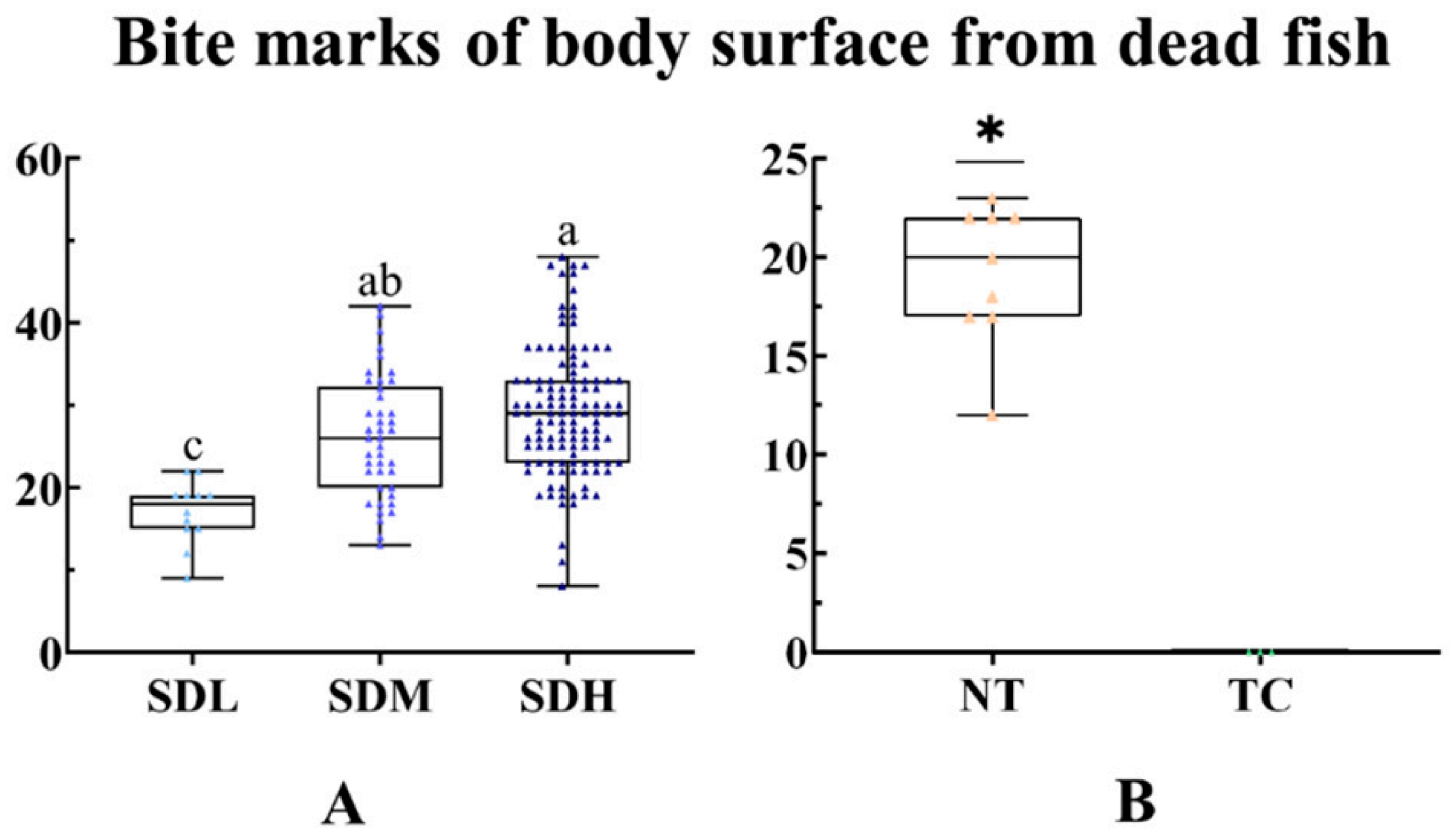
Bite marks of body surface from dead fish during the feeding trial. (**A**) is for the experiment of different stocking densities; (**B**) is for the experiment of tooth clipping. Tukey-style whiskers indicate minimum and maximum numbers of bite marks, while boxes indicate Q1, median, and Q3 values for interquartile range. Different letters are significant differences (Tukey’s test, *p* < 0.05) between treatments. “*” indicates significant differences (*t*-test, *p* < 0.05) between treatments.

Bite marks of body surface from surviving and dead fish are presented in Figure 6 and Figure 7, respectively. For the experiment testing different stocking densities, box plots showed that bite marks on body surface for both surviving (medians: SDL/SDM/SDH = 1/4/8) and dead fish (medians: SDL/SDM/SDH = 18/26/29) increased significantly (*p* < 0.05) with increasing initial stocking density. Similarly, for the experiment testing tooth clipping, bite marks of body surface for both surviving (medians: NT/TC = 2/0) and dead fish (medians: NT/TC = 20/0) were significantly (*p* < 0.05) higher in the NT group than in the TC group.

**Figure 8:**
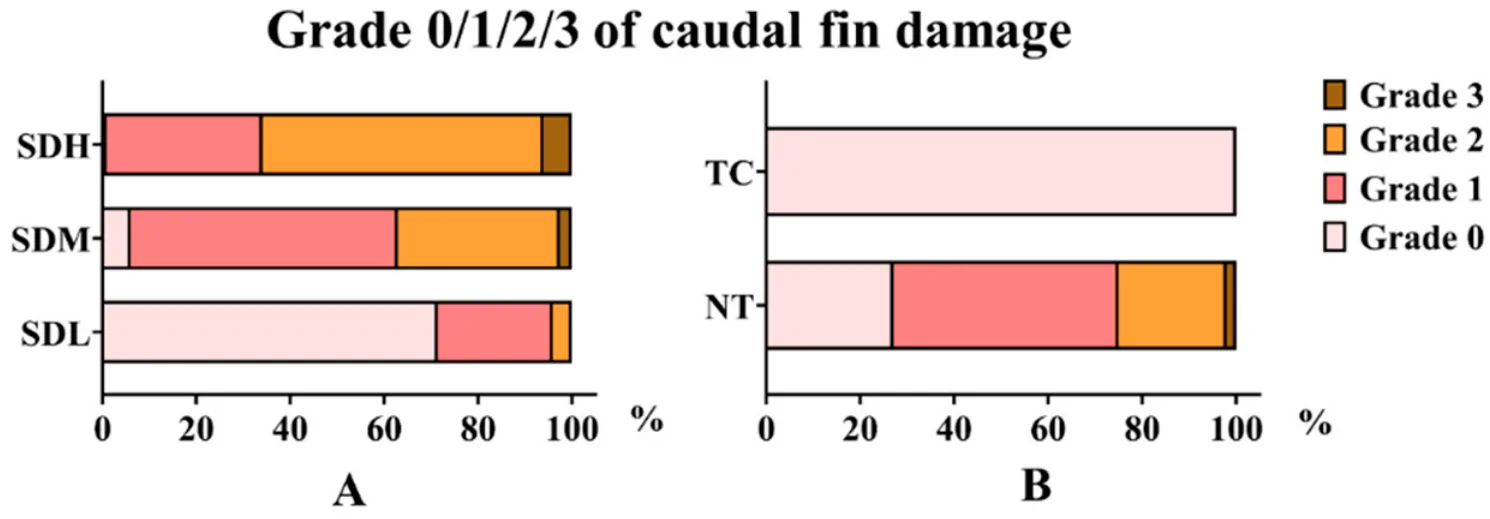
Grade 0/1/2/3 of caudal fin damage from surviving fish at the end of the feeding trial. (**A**) is for the experiment of different stocking densities; (**B**) is for the experiment of tooth clipping.

Caudal fin damage from surviving fish at the end of the feeding trial was classified into four categories (Grade 0/1/2/3) (Table 4 and Figure 8). For the experiment testing different stocking densities, the proportion of fish with no caudal fin damage (Grade 0) was 72.58 ± 6.31% in the SDL group, which was significantly (*p* < 0.05) higher than that in the SDM and SDH groups. The highest proportion of caudal fin damage in the SDM and SDH groups was Grade 1 and Grade 2, respectively. For the experiment testing tooth clipping, all fish in the TC group had a completely intact caudal fin (Grade 0), but only 26.30 ± 6.30% of fish in the NT group did. In addition, in both experiments, all dead fish exhibited caudal fin damage at Grade 2 and Grade 3; therefore, detailed grade-specific data were not presented here.

### 3.4. Indicators in Serum and Liver to Assess the Stress Challenge

**Figure 9:**
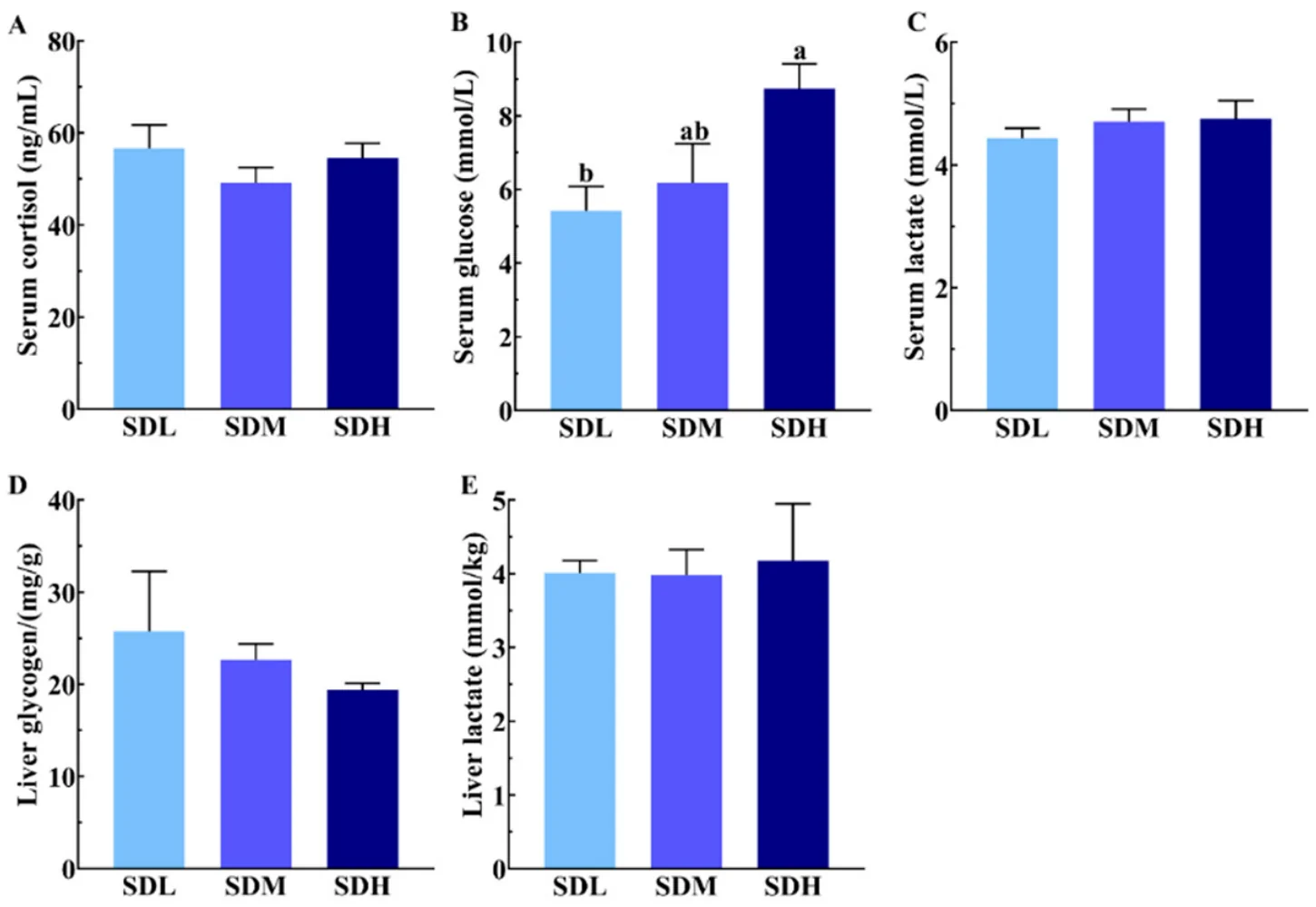
Indicators in serum and liver to assess the stress challenge for the experiment of different stocking densities. (**A**): Serum cortisol; (**B**): Serum glucose; (**C**): Serum lactate; (**D**): Liver glycogen; (**E**): Liver lactate. Values are presented as mean ± standard error of three replicate tanks. Different letters are significant differences (Tukey’s test, *p* < 0.05) between treatments.

**Figure 10:**
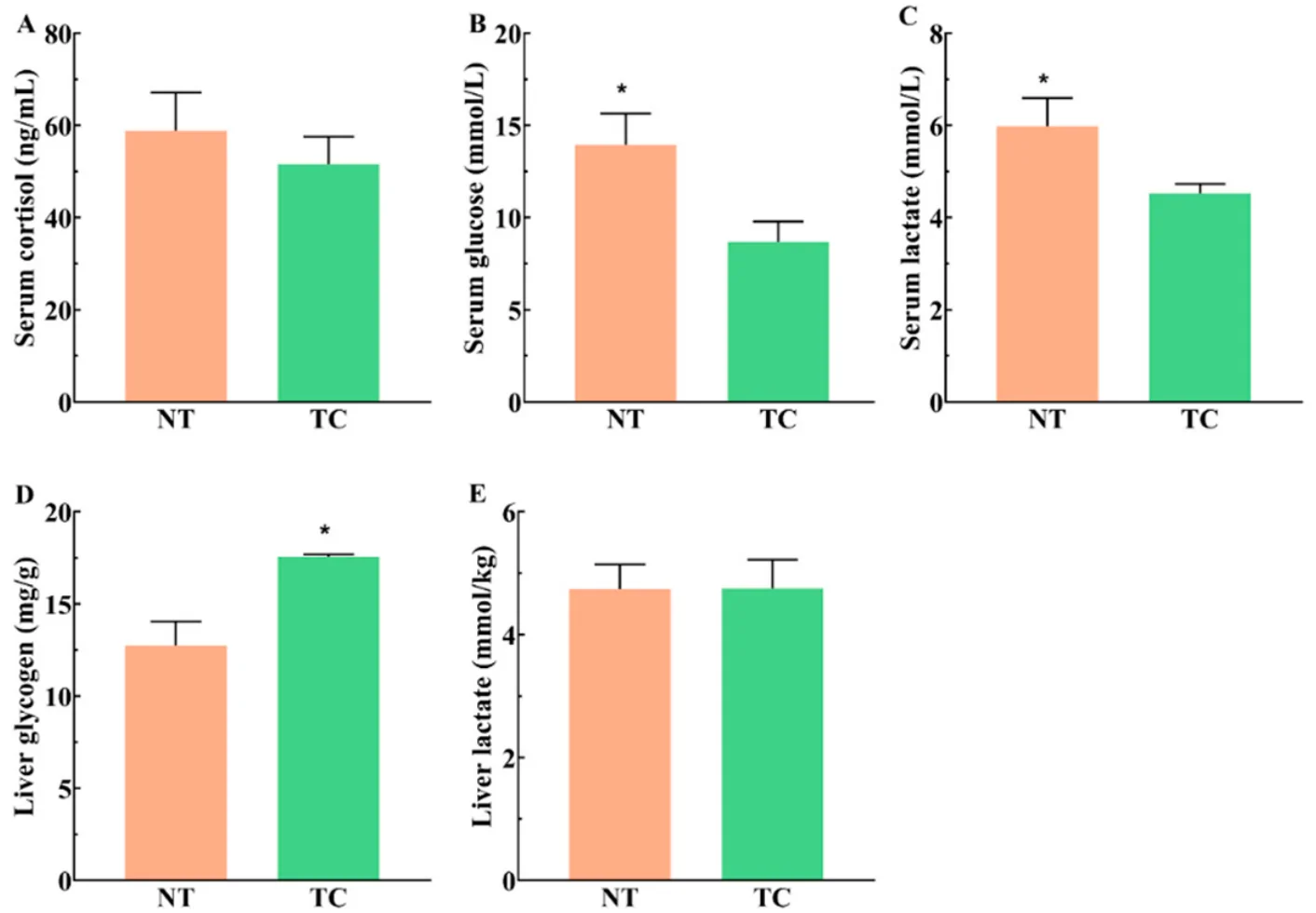
Indicators in serum and liver to assess the stress challenge for the experiment of tooth clipping. (**A**): Serum cortisol; (**B**): Serum glucose; (**C**): Serum lactate; (**D**): Liver glycogen; (**E**): Liver lactate. Values are presented as mean ± standard error of three replicate tanks. “*” indicates significant differences (*t*-test, *p* < 0.05) between treatments.

For the experiment testing different stocking densities, serum cortisol, glucose, and lactate concentrations, as well as liver glycogen and lactate concentrations, are shown in Figure 9. For the experiment testing tooth clipping, the same parameters are presented in Figure 10. Serum glucose level gradually increased with the increase in initial stocking density, and was significantly (*p* < 0.05) higher in the SDH group than in the SDL group. There were no significant (*p* > 0.05) differences in serum cortisol and lactate concentrations as well as liver glycogen and lactate concentrations among the SDL, SDM and SDH groups. In addition, compared to fish with intact teeth, tooth clipping significantly (*p* < 0.05) decreased serum glucose and lactate concentrations, and significantly (*p* < 0.05) increased liver glycogen concentration. No significant differences (*p* > 0.05) were observed in serum cortisol and liver lactate between the NT and TC groups.

### 3.5. The Metabolite Concentrations of 5-HT and Dopamine Metabolism in the Brain

**Figure 11:**
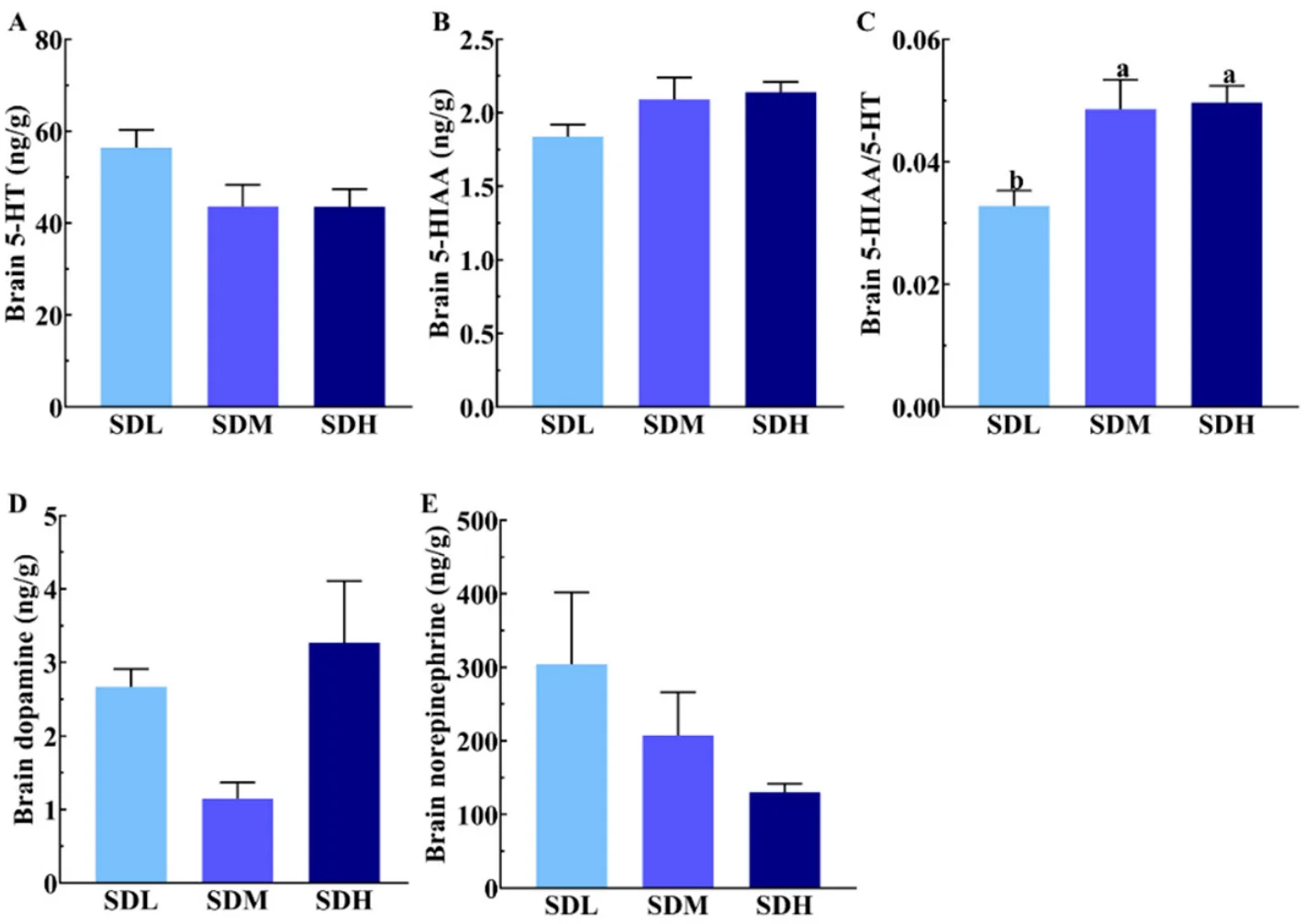
The metabolite concentrations of 5-HT and dopamine metabolism in the brain for the experiment of different stocking densities. (**A**): 5-HT; (**B**): 5-HIAA; (**C**): the ratio of 5-HIAA/5-HT; (**D**): dopamine; (**E**): norepinephrine. Values are presented as mean ± standard error of three replicate tanks. Different letters are significant differences (Tukey’s test, *p* < 0.05) between treatments. 5-HT: 5-hydroxytryptamine; 5-hydroxyindole acetic acid 5-HIAA.

**Figure 12:**
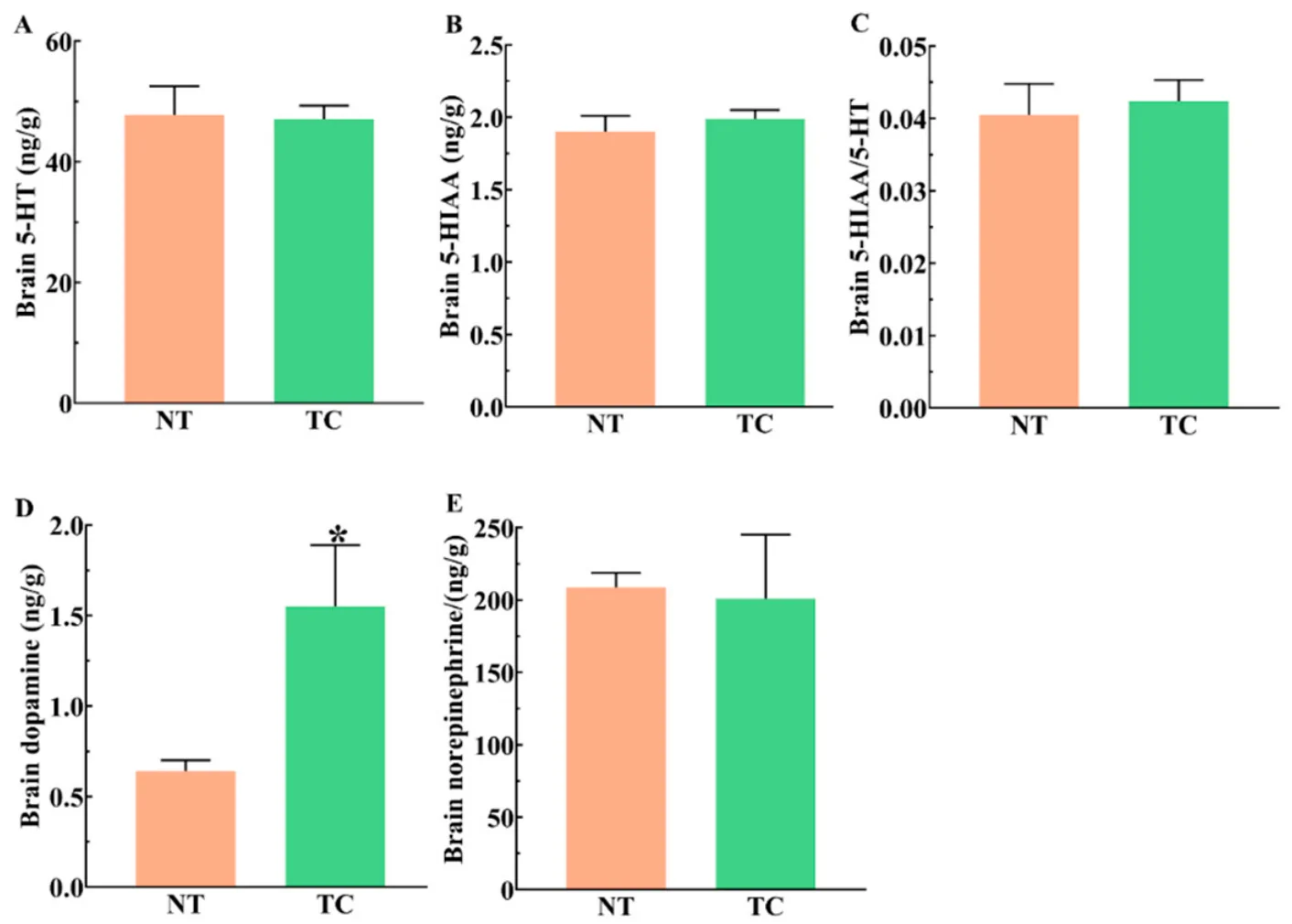
The metabolite concentrations of 5-HT and dopamine metabolism in the brain for the experiment of tooth clipping. (**A**): 5-HT; (**B**): 5-HIAA; (**C**): the ratio of 5-HIAA/5-HT; (**D**): dopamine; (**E**): norepinephrine. Values are presented as mean ± standard error of three replicate tanks. “*” indicates significant differences (*t*-test, *p* < 0.05) between treatments. 5-HT: 5-hydroxytryptamine; 5-hydroxyindole acetic acid 5-HIAA.

The metabolite concentrations of 5-HT metabolism (5-HT, 5-HIAA and the ratio of 5-HIAA/5-HT) and dopamine metabolism (dopamine and norepinephrine) in the brain are presented in Figure 11 and Figure 12. For the experiment testing different stocking densities, the ratio of 5-HIAA/5-HT in fish reared at medium and high stocking density (SDM and SDH) was significantly (*p* < 0.05) higher than that of fish reared at low stocking density (SDL). Brain norepinephrine level decreased with the increase in initial stocking density (*p* > 0.05). There were no significant differences (*p* > 0.05) in brain 5-HT, 5-HIAA and dopamine concentrations among the three different stocking densities. For the experiment testing tooth clipping, only brain dopamine levels were found to be significantly higher in the TC group than in the NT group.

### 3.6. Gene Expression of 5-HT and Dopamine Metabolism in the Brain

**Figure 13:**
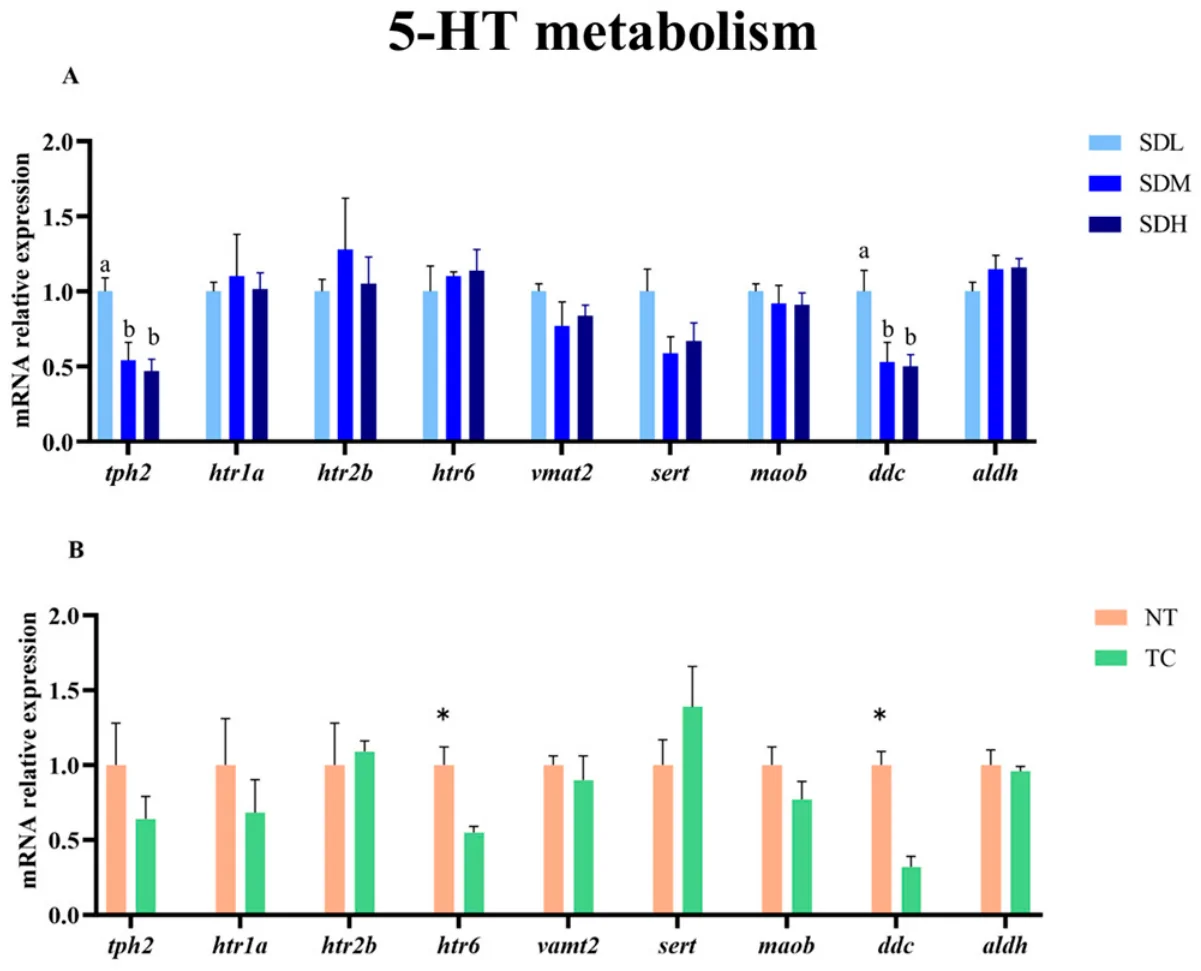
Gene expression related to the 5-HT metabolism in the brain for the experiment of different stocking densities. (**A**) is for the experiment of different stocking densities; (**B**) is for the experiment of tooth clipping. Values are presented as mean ± standard error of three replicate tanks. Different letters in (**A**) are significant differences (Tukey’s test, *p* < 0.05) among the SDL, SDM and SDH groups. “*” in (**B**) indicates significant differences (*t*-test, *p* < 0.05) between the NC and TC groups. 5-HT: 5-hydroxytryptamine; *tph2*: tryptophan hydroxylase 2; *htr1a*: 5- hydroxytryptamine receptor 1a; *vmat2*: vesicular monoamine transporter (SLC18A2); *sert*: serotonin transporter (SLC6A4); *maob*: monoamine oxidase B; *ddc*: aromatic L-amino acid decarboxylase; *aldh*: aldehyde dehydrogenase.

**Figure 14:**
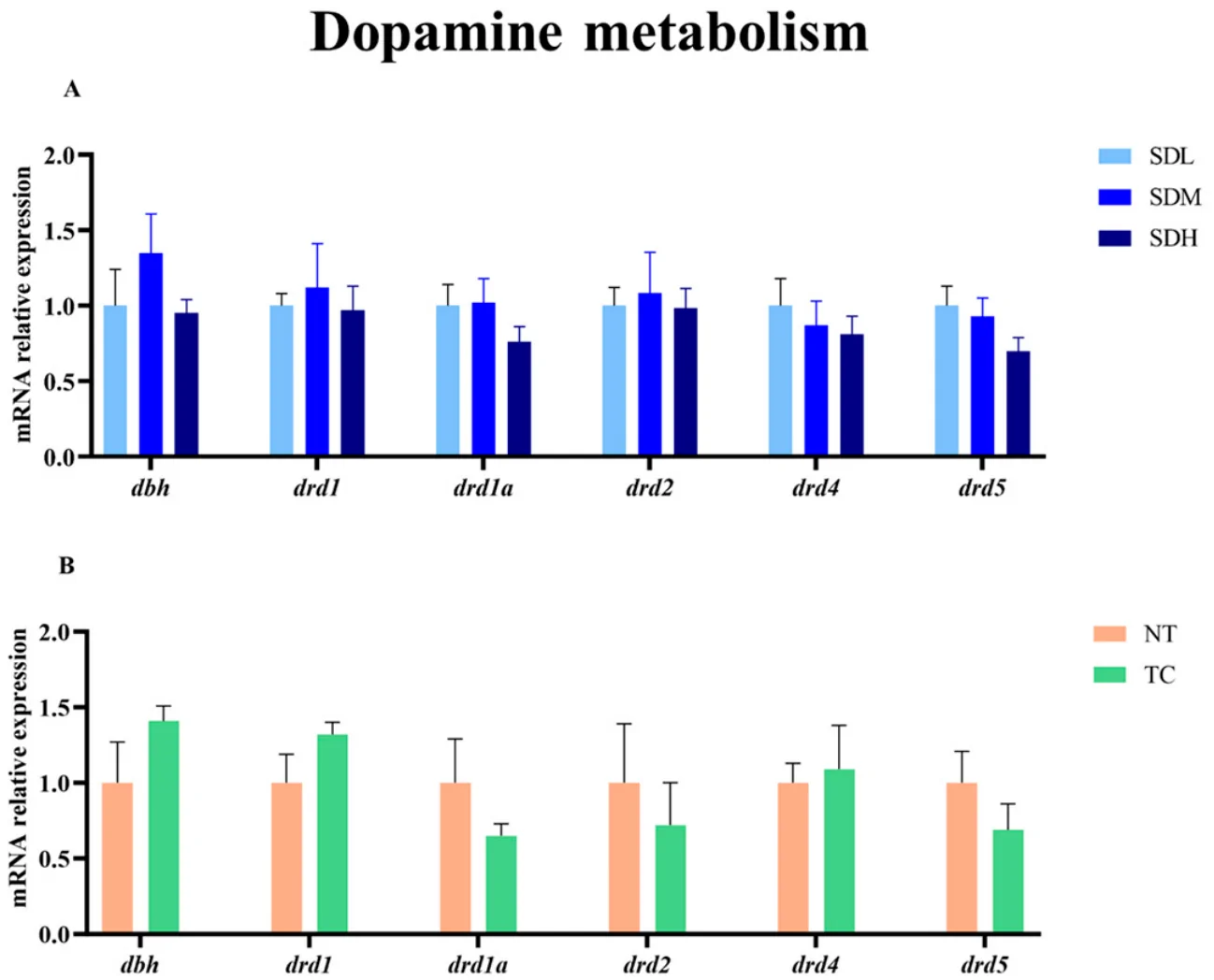
Gene expression related to dopamine metabolism in the brain. (**A**) is for the experiment of different stocking densities; (**B**) is for the experiment of tooth clipping. Values are presented as mean ± standard error of three replicate tanks. Different letters in (**A**) are significant differences (Tukey’s test, *p* < 0.05) among the SDL, SDM and SDH groups. *dbh*: dopamine beta-hydroxylase; *drd1*: D(1) dopamine receptor.

Gene expression related to 5-HT metabolism in the brain is presented in Figure 13. In 5-HT metabolism, the expression of genes including *tph2*, *htr1a*, *htr2b*, *htr6*, *vmat2*, *sert*, *maob*, *ddc* and *aldh* in the brain were determined. For the experiment testing different stocking densities, mRNA levels of *tph2* and *ddc* were significantly (*p* < 0.05) affected by dietary treatments, and they were significantly (*p* < 0.05) higher in the SDL group than in the SDM and SDH groups. For the experiment testing tooth clipping, the expression of *htr6* and *ddc* in the NT group was significantly (*p* < 0.05) higher than that of the TC group. Gene expression related to dopamine metabolism in the brain is presented in Figure 14. In the dopamine metabolism, the expression of genes including *dbh*, *drd1*, *drd1a*, *drd2*, *drd4* and *drd5* in the brain was determined. However, there were no significant (*p* > 0.05) differences in the expression of any determined genes for the experiments testing different stocking densities and tooth clipping.

## 4. Discussion

It is commonly recognized that stocking density has a significant impact on fish growth. Specifically, as the stocking density increases, the inhibitory effect on fish growth performance becomes more pronounced [10,29,30]. It was confirmed in this study, where the growth of tiger puffer in the initial high-stocking density group (80 fish per tank) was significantly lower than that in the initial low-stocking density group (20 or 40 fish per tank). In addition, it was notable that the treatment of tooth clipping promoted the growth of tiger puffer as a numerical trend, which is similar to our previous study [31], where the growth of tiger puffer with intact teeth is significantly reduced compared with those with clipped teeth when fed diets with an optimum level of tryptophan. This indicates that although tooth clipping is an additional stress in the culture of tiger puffer, its inhibition effect on growth may be relatively less severe compared to the intra-cohort cannibalism caused by the presence of intact teeth. Therefore, to reach maximum growth potential, tiger puffer farming requires an optimum stocking density, and tooth clipping at the beginning of culture is also advisable.

The factors influencing cannibalism in fish can be classified into two major categories: population and environmental factors. Population factors include genetic background, sex, fish size, stocking density and domestication; environmental factors include feed, light, water temperature and color, and tank shape [22]. As an environmental factor related to feed, feeding restriction has been proven to increase the severity of cannibalistic behavior in tiger puffer, and the most direct evidence is a significant reduction in survival rate [21,32]. In the natural environment, cannibalism is an important population regulating factor for fish [33]. Under rearing conditions, as a population factor, initial stocking density has been found to have an important impact on cannibalism in tiger puffer at the larvae stage, which can also be directly observed through survival rate [34,35]. Similarly, at the juvenile stage of tiger puffer, survival rate in this study indicated that low initial stocking density was effective in preventing mortality caused by the cannibalism. Meanwhile, tooth clipping treatment increased survival rate of juvenile tiger puffer from 83.33% to 95%. In addition, the inhibitory effect of reducing stocking density and tooth clipping on cannibalism was supported by observations of other parameters related to cannibalism. The gradation of caudal fin biting based on fin retained length is a useful tool for assessing the degree of cannibalism not only in lumpfish (*Cyclopterus lumpus*) [36,37], but also in tiger puffer [25]. Our findings show that at low stocking density, more than 95% of tiger puffer with intact teeth either did not have any damage in the caudal fin (Grade 0) or had only slight damage (Grade 1). Meanwhile, more promisingly, the combination of low rearing density and tooth clipping resulted in almost all tiger puffer having undamaged caudal fin. A round imprint nipped by fish teeth, termed a bite mark on the body surface, is another indicator for estimating the severity of cannibalism [38]. Similarly, both low stocking density and tooth clipping reduced the occurrence of bite marks on the body surface, whether in survival or dead fish. The current proof showed that the presence of intact teeth was the decisive internal factor causing caudal fin damage, and reducing the stocking density could effectively alleviate caudal fin biting. However, because the damaging effects of teeth biting the caudal fin or leaving bite marks on the body surface are weakened after tooth clipping, further study is needed to determine whether tooth clipping reduces the degree of cannibalism or the incidence of aggression in tiger puffer.

The behavior of fish is characterized by social interaction and hierarchies, such as aggressiveness and territoriality [39]. When fish are re-arranged into new groups, a social hierarchic order needs to be re-established. The establishment of social hierarchy among piscivorous fish may be accompanied by an increase in cannibalistic behavior [22,40]. In the present study, the two experiments differed in initial body weight, starting time, and rearing duration. However, during the first approximately 20 days after tiger puffer were distributed randomly among tanks, survival curve showed a rapid increase in mortality of all the treatments in both the different stocking densities and tooth clipping experiments. Tiger puffer have a territorial instinct and thus will bite each other as a result [41]. Therefore, the main reason for increased mortality during this period may not been due to stocking density, but rather increased cannibalistic behavior resulting from the re-establishment of social hierarchies after tiger puffer were stocked into new circular tanks. The formation time of social hierarchy seems to vary depending on the species of fish, apart from being influenced by the experimental conditions. Studies have reported that adult European sea bass (*Dicentrarchus labrax* L.) can establish a social hierarchic order within just one to two days [39]. However, in this study, based on survival curves over time, it was estimated that tiger puffer would take about 20 days to establish a social hierarchy order. In addition, social hierarchies in some fish species are caused by size variation [42,43]. However, considering the initially stocked fish in this study were kept at a similar size, this rules out the possibility that size heterogeneity caused the establishment of social hierarchies in tiger puffer. Other causes for social hierarchies, such as physiological factors, still need to be further investigated in tiger puffer [44,45].

There is evidence that cannibalistic behavior in tiger puffer triggers stress responses [46]. Some physiological parameters (cortisol, glucose, glycogen and lactate levels) are often used to monitor stress responses in fish. The increase in serum cortisol, glucose and lactate indicates that fish are undergoing stress responses [47,48]. This study found that as the stocking density increased, serum glucose level increased, indicating that high stocking density might be a potential source of chronic stress for tiger puffer [49]. Furthermore, for fish species like tiger puffer that exhibit typical cannibalistic behavior, intensified cannibalism caused by high stocking density may also indirectly increase the stress response. Different from the experiment testing different stocking densities, in fish with clipped teeth, stressors were relatively simple and caused only by cannibalism compared with fish with intact teeth. This may be one reason why changes in stress-related physiological indicators (lactate) were not consistent between the two experiments. In addition, in the liver, glycogen can be stored or depleted to maintain glucose homeostasis via glycogenesis or glycogenolysis [50,51]. In the experiment testing tooth clipping, liver glycogen level was higher in fish with clipped teeth than that in fish with intact teeth. This suggests that tiger puffer with intact teeth might require more energy than those with clipped teeth due to stress caused by cannibalism. Therefore, in order to maintain glucose homeostasis in the blood, stored liver glycogen is depleted to produce glucose, which serves as energy for other tissues. Changes in serum lactate and liver glycogen seemed to suggest that stress responses caused by cannibalism due to intact teeth in tiger puffer may be more obvious than those caused by high stocking density.

In fish and many other animals, cannibalistic or aggressive behaviors are linked to neurotransmitters, particularly 5-HT and dopamine [52,53,54,55]. The brain dopaminergic system seems to have a stimulating effect on aggressive behavior in fish, whereas the brain serotonergic system seems to have an inhibiting effect [56]. Concentrations of 5-HT and its metabolite 5-HIAA have been used as indicators for investigating the aggression in some experimental animals [57]. However, the effects of 5-HT concentration on cannibalistic or aggressive behaviors are still paradoxical. Although most studies support the argument that increasing 5-HT levels can inhibit cannibalistic or aggressive behavior in fish, reptiles, and mammals [58], other studies have found an increased 5-HIAA/5-HT ratio in specific brain regions of winning or losing fish [59]. In this study, the ratio of 5-HIAA/5-HT was significantly elevated in the high stocking density treatment, which was the treatment with severe cannibalism in tiger puffer. Additionally, it was found that different stocking densities affected the expression of *tph2* and *ddc* related to 5-HT metabolism. These collectively indicated that cannibalistic behavior induced by high stocking density was associated with 5-HT metabolism. Interestingly, our previous study found that increased tryptophan levels coincided with a decreased ratio of 5-HIAA/5-HT [31], which supports that tryptophan supplementation in diets can alleviate cannibalistic behavior by regulating 5-HT metabolism. However, it must be acknowledged that when measuring neurotransmitter concentrations, the accuracy of results obtained from measuring the entire brain region is lower than that obtained from measuring specific localized areas of the brain [53]. It may explain why no alteration in the ratio of 5-HIAA/5-HT was observed in the tooth clipping experiment. Regarding dopamine metabolism, this study showed that dopamine level in fish with clipped teeth was significantly higher than that of fish with intact teeth. In various species, elevated dopamine levels have been considered to be associated with increased aggressive behavior [54,60,61,62]. However, after tooth clipping in this study, cannibalistic behavior in tiger puffer was alleviated, as evidenced by survival rate, bite marks of body surface and caudal fin damage. In the tooth clipping treatment, the reduction in cannibalistic behavior was not accompanied by the expected decrease in dopamine levels. A possible explanation is that while tooth clipping reduced cannibalistic behavior, it may not have suppressed the motivation for aggression in tiger puffer, which may have even increased. The observed reduction in cannibalistic behavior could be due to tooth clipping reducing the damage caused by attacks [63]. Nevertheless, this is only a speculative hypothesis that requires confirmation in further studies.

In this study, several limitations should be acknowledged. First, the experimental design testing tooth clipping lacked a sham-operated control, the NT group received only air exposure, meaning that handling-specific effects cannot be fully separated from clipping-specific effects. Second, teeth regrowth was not monitored after the procedure, so clipping effect duration across 48 days is unknown, and potential regrowth may have diluted the treatment effect over time. Third, our neurochemical analyses were performed on whole-brain homogenates, which inevitably lose regional resolution. Region-specific assays (hypothalamus, telencephalon) would better localize 5-HT changes. Fourth, with only three replicate tanks per treatment (*n* = 3), the statistical power for growth and survival was inherently limited. Therefore, the non-significant *p*-values for these parameters should not be interpreted as evidence of no effect. Fifth, this study was conducted at the single life stage (the juvenile stage with initial body weight of approximately 7–24 g), and it remains unclear whether larvae or pre-adult stages exhibit similar responses to stocking density or tooth clipping. Finally, cannibalistic behavior was inferred from post hoc physical damage (bite marks and caudal fin grading) rather than from direct video observation of attack frequency, which would be needed to quantify attack frequency and establish causality more robustly.

## 5. Conclusions

The internal factor underlying cannibalistic behavior in tiger puffer is the presence of sharp teeth, and tooth clipping alleviates cannibalism and tends to improve growth. Additionally, during the juvenile stage of tiger puffer, increasing the initial stocking density is another population factor contributing to the exacerbation of cannibalism. In addition to stress response, stocking density-induced cannibalism is related to 5-HT metabolism, whereas clipping teeth is associated with increased brain dopamine level. However, whether this means that clipping teeth can only mitigate the degree of damage caused by cannibalism and cannot reduce the motivation for aggression remains to be further verified. Furthermore, according to results of the Kaplan–Meier survival curve, it appears that tiger puffer may need about 20 days to re-establish the social hierarchy order when being relocated to a new rearing environment. However, this inference is derived from survival curves, and it still requires direct behavioral records (e.g., bite frequency over time) to confirm this hypothesis.

## Figures and Tables

**Table 1:** Primer sequences used for qRT-PCR.

Gene	Primer Sequence (5′-3′)	Product Size (bp)	Genbank No. ^1^
*tph2*	F: AGAGGGATGTCTCTGGACTCG	90	NM_001032677.1
	R:AATGGGAGAGAAGGACGGAC		
*htr1a*	F:TCTACTCCACTTTCGGGGCT	154	XM_011620867.2
	R:GCCGGAGATAAAGTCAGGCA		
*htr2b*	F:CCATACCGTGTCCTTGCCTT	182	XM_029828249.1
	R:GCTCCAGCCCAGGATTTTCT		
*htr6*	F:GGCTGTGATTGTGCATGGTT	183	XM_029833408.1
	R:GCCTGCTTTCATAAGTGCCC		
*vmat2*	F:AGGGTGTTGGTTCATCCTGC	158	XM_029835537.1
	R:TTCATACATCACGCTGCCGA		
*sert*	F:GGAGAAACTACTCAGCACGAC	202	XM_029843335.1
	R:GCCAAGCACGGTAAAGAT		
*maob*	F:CCCGAGTGCTTGGAGATACC	82	XM_003961571.3
	R:TCAGGAAACCACCAACGGAG		
*ddc*	F:GAACAGCGTCCAGTCTACCC	245	XM_003965983.3
	R:GCCCAAGAGAAGCCGATACA		
*aldh*	F:TACGGAGGTCAAGACGGTGA	76	XM_003965850.3
	R:GGTTTCTCGAAGGTCGGTCA		
*dbh*	F:TCGGTATCGTTTGTCGTCGT	163	XM_029837394.1
	R:GCCGCTGTTTGACCTGAAAG		
*drd1*	F:TCGGTATCGTTTGTCGTCGT	155	XM_003970533.3
	R:TCATCACGTAAGCCACTCGG		
*drd1a*	F:CAGCGTCATCATGGGTGTCT	164	XM_029851504.1
	R:AGTTGCTCCATCCGATCCAC		
*drd2*	F:AAACGAGTGTCAAGCAGGGA	249	XM_029839446.1
	R:AGCAGGAAGAAATCTGGTGAGT		
*drd4*	F:CAGTTCACTTACGCGCTGTG	99	XM_029833683.1
	R:CTCGGTCTGAAAGGGGAAGG		
*drd5*	F:CGCTGAGGGTACTCTGGTTG	187	XM_003973551.2
	R:GCGTGGAGCGTAAAACAGAC		
*rpl13*	F:GTAACAGGTCCACAGAATCCC	117	XM_011610267.2
	R:CCTCAGTGCTGTCTCCCTTC		
*rpl19*	F:GATCCCAACGAGACCAACGA	191	XM_003964816.3
	R:CGAGCATTGGCTGTACCCTT		

Abbreviations: *tph2*: tryptophan hydroxylase 2; *htr1a*: 5- hydroxytryptamine receptor 1a; *vmat2*: vesicular monoamine transporter (SLC18A2); *sert*: serotonin transporter (SLC6A4); *maob*: monoamine oxidase B; *ddc*: aromatic L-amino acid decarboxylase; *aldh*: aldehyde dehydrogenase; *dbh*: dopamine beta-hydroxylase; *drd1*: D (1) dopamine receptor; *rpl13*: ribosomal protein L13; *rpl19*: ribosomal protein L19. ^1^ NCBI GenBank accession no.

**Table 2:** Growth and body condition indices of the experiment of different stocking densities ^1^.

	SDL	SDM	SDH
Initial body weight (g)	7.18 ± 0.12	6.95 ± 0.13	6.96 ± 0.09
Final body weight (g)	72.40 ± 3.03 ^a^	66.65 ± 2.30 ^a^	55.08 ± 2.34 ^b^
Weight gain (%)	909.36 ± 45.43 ^a^	858.91 ± 31.43 ^a^	692.57 ± 39.99 ^b^
Specific growth rate (%/d)	3.00 ± 0.06 ^a^	2.93 ± 0.04 ^a^	2.68 ± 0.07 ^b^
Viscerosomatic index (%)	16.36 ± 0.29 ^a^	16.29 ± 0.25 ^a^	14.13 ± 0.42 ^b^
Hepatosomatic index (%)	12.68 ± 0.31 ^a^	13.40 ± 0.39 ^a^	11.44 ± 0.15 ^b^

^1^ Values are presented as means and standard error of three replicate tanks. Values in the same row followed by different superscript letters are significantly different (Tukey’s test, *p* < 0.05) between treatments.

**Table 3:** Growth and body condition indices of the experiment of tooth clipping ^1^.

	NT	TC
Initial body weight (g)	24.28 ± 0.16	24.21 ± 0.26
Final body weight (g)	71.76 ± 2.54	80.82 ± 3.33
Weight gain (%)	195.57 ± 11.82	234.18 ± 19.26
Specific growth rate (%/d)	1.45 ± 0.05	1.56 ± 0.08
Viscerosomatic index (%)	16.74 ± 0.56	16.20 ± 0.31
Hepatosomatic index (%)	12.66 ± 0.60	11.94 ± 0.27

^1^ Values are presented as means ± standard error of three replicate tanks. No significant differences (*t*-test, *p* < 0.05) were recorded between treatments.

**Table 4:** Grade 0/1/2/3 of caudal fin damage from surviving fish at the end of the feeding trial ^1^.

	Grade 0	Grade 1	Grade 2	Grade 3
The experiment of different stocking densities				
SDL	72.58 ± 6.31 ^a^	23.19 ± 6.48 ^b^	4.23 ± 1.53 ^c^	0
SDM	6.10 ± 3.07 ^b^	56.65 ± 5.74 ^a^	34.65 ± 6.67 ^b^	2.59 ± 1.70
SDH	0.95 ± 0.55 ^b^	33.26 ± 4.30 ^b^	59.67 ± 4.28 ^a^	6.12 ± 2.63
The experiment of tooth clipping				
NT	26.30 ± 6.30	47.78 ± 4.01 *	23.70 ± 6.58	2.22 ± 2.22
TC	100 *	0	0	0

^1^ Values are presented as means ± standard error of three replicate tanks. Values in the same column followed by different superscript letters are significantly different (Tukey’s test, *p* < 0.05) between treatments. Values in the same column followed by “*” are significantly different (*t*-test, *p* < 0.05) between treatments.

## Data Availability

Data can be made available on request.

## Supplementary Materials

The following supporting information can be downloaded at https://www.mdpi.com/article/10.3390/biology15191766/s1, Table S1: Ct values of both *rpl13* and *rpl19* across treatments.
