# Supplementary material for: Factors Influencing Cannibalistic Behavior of Tiger Puffer, Takifugu rubripes: Insights into Teeth and Stocking Density Aspects
Source: Biology (Basel). 2026 Oct 4;15(19):1766. doi: 10.3390/biology15191766 (PMC13664422; doi:10.3390/biology15191766)
Supplement: Supplementary file 1 [file biology-15-01766-s001.zip › biology-4558240-supplementary.pdf]

Table S1 Ct values of both *rpl13* and *rpl19* across treatments.

	rpl13	*rpl19*
The experiment of different stocking density		
SDL-1	17.83	18.29
SDL-2	18.61	19.17
SDL-3	17.95	18.39
SDM-1	17.41	17.40
SDM-2	17.91	18.37
SDM-3	18.45	19.30
SDH-1	18.22	18.72
SDH-2	18.02	18.54
SDH-3	18.10	18.85
The experiment of teeth clipping		
NT-1	19.10	19.89
NT-2	19.94	20.37
NT-3	20.90	22.35
TC-1	19.90	20.74
TC-2	20.47	21.21
TC-3	18.66	18.86
